# Antibiotic treatment of *Clostridium difficile* infection in children – a challenge in pediatric practice

**DOI:** 10.1186/1471-2334-14-S7-P6

**Published:** 2014-10-15

**Authors:** Gabriela Leşanu, Cristina Becheanu, Raluca Maria Vlad, Daniela Lemeni, Ioana Sabina Macovei, Daniela Păcurar, Mirela Stocklosa, Irina Andronie, Alexandra Moraru

**Affiliations:** 1 Grigore Alexandrescu Clinical Children’s Emergency Hospital, Bucharest, Romania; 2 Cantacuzino National Institute for Research and Development for Microbiology and Immunology, Bucharest, Romania

## Background

In the last decade the incidence of *Clostridium difficile* infection (CDI) in children is progressively increasing and the pediatricians are faced with difficulties in the therapeutic approach.

## Methods

We performed a retrospective study that analyzed the antibacterial treatment in CDI from the experience of a Pediatric Gastroenterology Department – Grigore Alexandrescu Clinical Children’s Emergency Hospital, Bucharest. Cases were identified through enzyme immunoassays for A toxin or for A and B toxin of *Clostridium difficile* in the stool.

## Results

Between January 1^st^ 2005 and July 31^st^ 2014, 52 patients were diagnosed with CDI. A large number of cases (61%) were diagnosed in the age group 1 to 4 years. The sex ratio was M/F = 0.9/1. 36% of patients had community-acquired CDI. In mild/moderate forms metronidazole was administered as a first-line treatment in 32 (61%) cases and proved efficient in 25/32 (78.1%) cases; vancomycin was used and was efficient in 18 cases. In severe forms (7.7%), the association of intravenous metronidazole and oral vancomycin was the option of choice and this approach cured all these cases. We report 11 patients with recurrent CDI (21%); in these cases oral vancomycin was efficient for the treatment of the recurrence. In 3 cases with a second recurrence rifaximin was the chosen therapy. Six out of 11 children with recurrent CDI had comorbidities (Hirschsprung disease, ulcerative colitis).

## Conclusion

The majority of patients in the study group were 1 to 4 years aged children. One third of patients had community-acquired CDI. We consider that the failure rate for metronidazole treatment is small and thereby metronidazole may be recommended for the treatment of the first episode of mild/moderate CDI. The association of intravenous metronidazole and oral vancomycin remains the treatment of choice for severe cases.

